# Mindfulness Meditation and Tai Chi Chuan on Sleep Disturbance in Chinese Older People: A Randomized Controlled Trial

**DOI:** 10.1093/geroni/igaa057.610

**Published:** 2020-12-16

**Authors:** Sunny Chan, Wai Chi Chan, Siu Man Ng, Chong Ho Alex Yu

**Affiliations:** 1 The Hong Kong Polytechnic University, Hong Kong, China; 2 The University of Hong Kong, Hong Kong, China; 3 Azusa Pacific University, Azusa, California, United States

## Abstract

Sleep disturbances are common during the aging process and can result in a reduced quality of life. Many older people who experience sleep disturbances would consider turning to complementary and alternative medicine (CAM) due to the limitations of traditional pharmaceutical or psychological and behavioural treatments. Mindfulness Meditation (MM) and Tai Chi Chuan (TCC) are two common forms of mind-body based CAM. The former focuses more on mind-based practices whereas the latter emphasizes predominantly on body or movement-based practices. An etiological model of sleep disturbance (Shallcross et al., 2019) can lay the groundwork for a better understanding of the mechanisms of MM and TCC in relation to sleep disturbances. This study aims at comparing the effects of MM and TCC with Sleep Hygiene Education (SHE) control group. A three-armed randomized controlled pilot trial was conducted involving 45 community-dwelling older adults aged 65 to 82 with symptoms of sleep disturbance. Moderate effect sizes (Cohen’s d = 0.7 and 0.56) were found for the primary outcome of insomnia severity at post-intervention as comparing MM and TCC groups with SHE control group, respectively. More specific, participants in the MM group showed more amelioration on mental health status, introspective awareness, and objective measure of EEG-based brain arousal level; whereas participants in the TCC group showed better improvement on physical health status and subjective measure of hyperarousal. Findings demonstrate the unique therapeutic effects of MM and TCC on improving sleep problem in older people. The application in a Chinese context will be discussed.

